# Increased nerve growth factor expression and osteoclast density are associated with subchondral bone marrow lesions in osteoarthritic knees

**DOI:** 10.1016/j.ocarto.2024.100504

**Published:** 2024-07-23

**Authors:** Koji Aso, Natsuki Sugimura, Hiroyuki Wada, Syo Deguchi, Masahiko Ikeuchi

**Affiliations:** Department of Orthopedic Surgery, Kochi Medical School, Kochi University, 185-1 Oko-cho Kohasu, Nankoku 783-8505, Japan

**Keywords:** Knee osteoarthritis, Pain, Bone marrow lesion, Nerve growth factor, Osteoclast

## Abstract

**Objectives:**

Subchondral bone marrow lesions (BMLs) detected on magnetic resonance imaging in knee osteoarthritis (OA) are associated with knee pain, though the mechanisms remain unknown. Increased nerve growth factor (NGF) expression and osteoclast density in subchondral bone appear to be the key features associated with bone pain in knee OA. Therefore, we aimed to identify associations among NGF, osteoclasts, and BMLs in knee OA.

**Methods:**

Twenty tibial plateaus were obtained from patients undergoing total knee arthroplasty for medial knee OA with BMLs at the medial tibial plateau (MTP). Osteochondral tissue samples from the weight-bearing part of the MTP, with and without BML, and from the weight-bearing part of the lateral tibial plateau (LTP), without BML, were collected. NGF expression and density of osteoclasts were compared among the three osteochondral tissue types.

**Results:**

MTP bone with BMLs exhibited significantly higher NGF expression in bone marrow space and osteochondral channel, and higher osteoclast density than MTP bone without BML and LTP bone. The mean differences in NGF-positive area in the bone marrow space and the percentage of NGF-positive channels between MTP bones with and without BML were 9.0% (95% confidence interval [CI]: 5.9–12.1%) and 23.1% (95% CI: 11.3–35.0%), respectively. The difference in osteoclast density between MTP bones with and without BML was 0.6 osteoclasts per mm (95% CI: 0.3–0.9 osteoclasts per mm).

**Conclusions:**

Increased NGF expression and osteoclast density are associated with subchondral BMLs in knee OA, contribute to understanding the mechanisms underlying BML-related bone pain in knee OA.

## 1 Introduction

Knee pain is the most common symptom and reason for seeking medical care in patients with knee osteoarthritis (OA). Knee OA is characterized by aberrations in the structural integrity of the joint including cartilage degeneration, synovial inflammation, and subchondral sclerosis with osteophyte formation. Recent clinical evidence [[1], [2], [3]] indicates that the subchondral bone is involved in the development of joint pain in OA. On magnetic resonance imaging (MRI), patients with knee OA exhibited subchondral marrow lesions (BMLs) more frequently than control patients without arthritis [1]. Subchondral BMLs are characterized by ill-defined hypointensity on T1-weighted, non-fat-suppressed images and hyperintensity on fluid-sensitive, T2-weighted, proton density-weighted, intermediate-weighted, fat-suppressed, and short tau inversion recovery images [4,5]. Subchondral BMLs are strongly associated with knee pain. Specifically, larger baseline subchondral BMLs were associated with greater baseline knee pain, and higher total subchondral BML volume was associated with increased knee pain [3]. The presence or size of BMLs can predict future BML incidence and progressive pain [6]. For varus knee OA, we have previously demonstrated that subchondral BML size in the medial femorotibial joint compartment was associated specifically with weight-bearing pain (rather than non-weight-bearing pain), more so than any other OA-related MRI features, such as effusion-synovitis, Hoffa's synovitis, cartilage defects, osteophytes, meniscus extrusions, anterior cruciate ligament tears, or age, sex, or body mass index [7].

The exact pathophysiology of subchondral BMLs is still subject to debate, and the cellular and molecular factors that mediate the association between subchondral BMLs and knee OA pain remain unknown. Subchondral BMLs are histologically characterized by bone marrow necrosis, trabecular abnormalities, bone marrow fibrosis, edema, cellular infiltration, and vascular proliferation [8,9]. Microarray analysis of subchondral BMLs in OA has revealed the upregulation of genes implicated in neurogenesis, osteochondral turnover, and inflammation [9]. We recently demonstrated that subchondral pathology is associated with knee OA pain independent of chondropathy and synovitis and that increased nerve growth factor (NGF) expression at the osteochondral junction and increased osteoclast density are key features associated with bone pain in knee OA [10]. Subchondral BMLs may contribute to OA pain via the generation of chemical factors that sensitize the nerves within the subchondral bone [11]. Therefore, we hypothesized that elevated NGF expression and osteoclast density are associated with BMLs in knee OA. A recent histological study reported an increase in the tartrate-resistant acid phosphatase (TRAP)-positive cells and the expression of general neuronal marker protein-encoding gene product 9.5 (PGP9.5) in samples comprising subchondral BMLs combined with cysts [12]; however, the results of that study were influenced by systemic differences in bone metabolism, as the study compared individuals with and without BMLs.

Therefore, in this study, we aimed to identify the associations among NGF expression, osteoclast density, and subchondral BMLs in symptomatic knee OA, by comparing subchondral bone with and without BML in the same individual.

## 2 Patients and methods

### 2.1 Patient samples

**Fig. 1:**
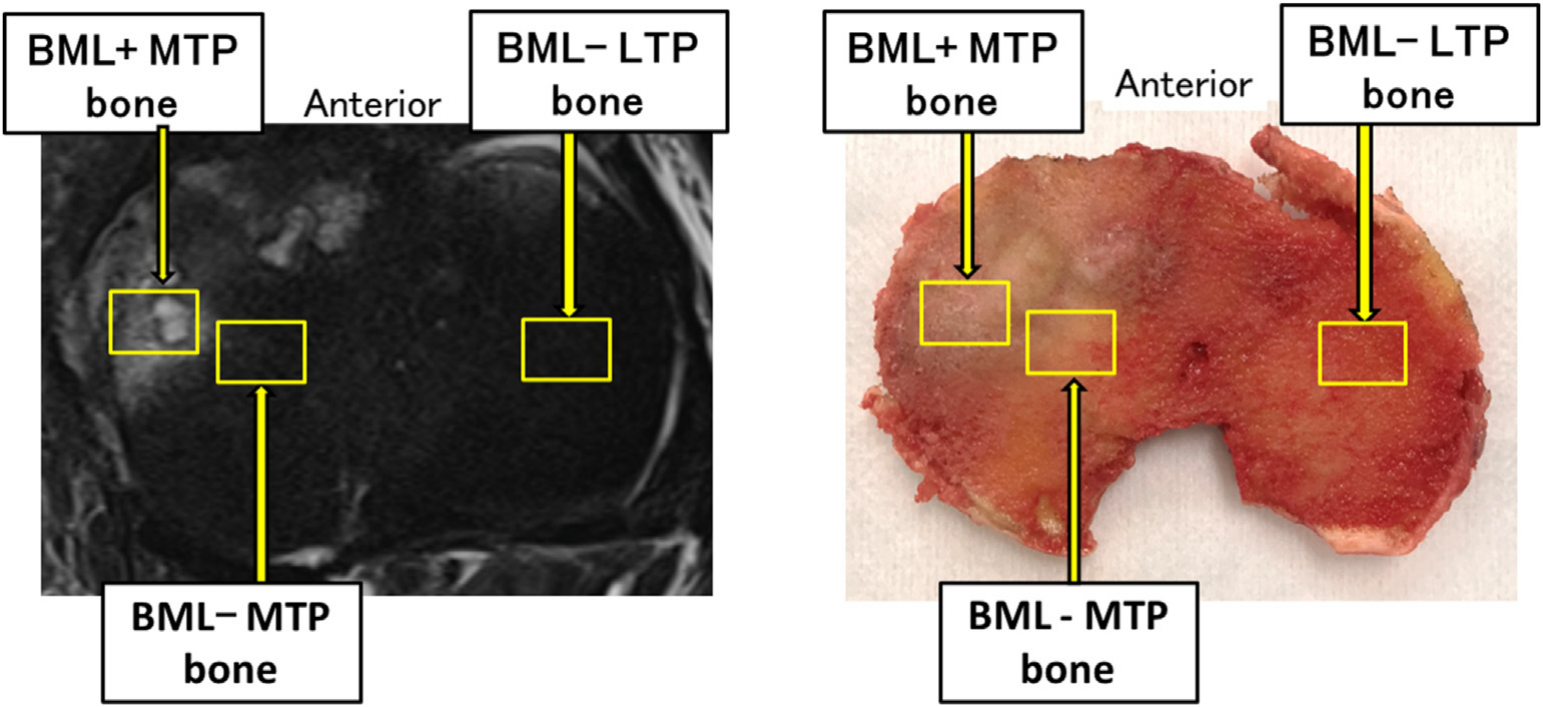
**Co-localization of MRI Images and joint tissue obtained at surgery**. Representative images of axial views of target knee for co-localization of knee biopsy tissue by MRI identification before tissue harvest at joint replacement surgery. Yellow boxes represent BML^+^ MTP bone, BML^−^ MTP bone and BML^−^ LTP bone that were analyzed. MTP: medial tibia plateau, LTP: lateral tibial plateau.

To identify associations among NGF, osteoclasts, and BML in knee OA, tibial plateaus were obtained from patients undergoing total knee arthroplasty (TKA) for medial knee OA with BMLs in the medial tibial plateau (MTP). Patients were recruited between August 2019 and September 2020. The exclusion criteria were as follows: insufficient amount of osteochondral tissue for evaluation; lateral knee OA; medial knee OA without BML; use of medication for osteoporosis; neuropathic arthropathy; psychological disorders; and inability to fill in the questionnaires. Twenty patients with medial knee OA with BMLs undergoing primary TKA were included in this study. We collected osteochondral tissue of approximately 1 ​× ​1.5 ​cm in size with and without BML from the weight-bearing part of the MTP and osteochondral tissue without BML from the weight-bearing part of the lateral tibial plateau (LTP) (Fig. 1). Written informed consent was obtained from all patients, and the protocols were approved by the Kochi University Research Ethics Committee (IRB:31–74).

### 2.2 Magnetic resonance imaging (MRI)

The knees were scanned using a 1.5T MRI machine with a knee coil (SIGNA Architect 3.0T; GE Healthcare, Chicago, IL, USA). The imaging sequences included sagittal, coronal, and axial T2-weighted fat-saturated fast spin echo (repetition time: 2500 ​ms, echo time: 90 ​ms, slice thickness: 3 ​mm, gap between slices: 1.0 ​mm). Bone marrow lesions (BML) were identified as areas of irregular hyperintense signal in the subchondral bone on the fat-saturated T2-weighted images (Fig. 1) [4]. The MRI scan was performed within one month before total knee arthroplasty (TKA)

### 2.3 Sample processing

Osteochondral tissues were fixed in neutral-buffered formalin and then decalcified by immersing them in Osteosoft solution® (Sigma-Aldrich Japan G.K) for three months at 4 ​°C before being embedded in paraffin wax. Formalin-fixed tibial plateau sections (5 ​μm) were stained with hematoxylin and eosin or Safranin O–fast green.

### 2.4 Histology and grading

Subchondral bone changes were graded using the osteoarthritis bone score [13]: cyst (0–1, where 0 ​= ​none and 1 ​= ​present), fibrosis (0–1, where 0 ​= ​none and 1 ​= ​present), blood vessels (0–1, where 0 ​= ​normal [0–15] and 1 ​= ​elevated [>16]), cartilage islands (0–1, where 0 ​= ​none and 1 ​= ​present), trabeculae thickened (0–1, where 0 ​= ​normal and 1 ​= ​elevated), tidemark integrity (0–1, where 0 ​= ​intact and 1 ​= ​crossed by at least one blood vessel), and inflammation (0–1, where 0 ​= ​absent and 1 ​= ​present). OA articular cartilage changes were graded using the OARSI OA cartilage histopathology assessment system [14] and Mankin scale [15]. The OARSI system follows the following grading system: Grade 0: surface intact, cartilage morphology intact; Grade 1: surface intact, superficial fibrillation, and edema or cell death or cell proliferation; Grade 2: surface discontinuity; Grade 3: vertical fissures (clefts); Grade 4: erosion; Grade 5: denudation; Grade 6: deformation. The Mankin scales are as follows: cartilage surface integrity (0–6, where 0 ​= ​normal and 6 ​= ​complete disorganization), tidemark integrity (0–1, where 0 ​= ​intact and 1 ​= ​crossed by vessels), chondrocyte morphology (0–3, where 0 ​= ​normal and 3 ​= ​hypocellular), and proteoglycan loss (0–4, where 0 ​= ​normal, no loss of Safranin-O stain, and 4 ​= ​complete loss of stain). Subchondral osteosclerosis was histologically assessed using trabecular bone volume/total volume, and subchondral bone plate thickness (μm) was quantified using computer-assisted image analysis (Keyence, Japan). Channel density in the osteochondral junction was calculated per millimeter.

### 2.5 Immunohistochemistry for NGF

Sections were subjected to antigen retrieval (10 ​mM citrate buffer, 90 ​°C, 20 ​min) and were blocked with 5% bovine serum albumin containing goat serum, followed by incubation with rabbit monoclonal antibody against NGF (EP1320Y, Abcam, Cambridge, UK) and biotinylated goat anti-rabbit IgG secondary antibody (BA1000, Vector, Peterborough, UK). Immunoreactivity to NGF was visualized using avidin-biotin-peroxidase complex (Vector, Peterborough, UK) with nickel-enhanced diaminobenzidine development [15]. The sections were counterstained with hematoxylin so that different regions were more apparent. The areas positive for NGF immunoreactivity in the subchondral bone were quantified. Briefly, the regions of interest (ROIs) in the subchondral bone were manually outlined. Positive staining was differentiated from the background by thresholding the image using hues to create a mask. Areas of positive staining and ROIs were measured. The fractional area was determined as the percentage of the area positive for NGF immunoreactivity within the ROI (Supplementary Materials). The proportion of NGF-positive channels at osteochondral junctions was measured.

### 2.6 Tartrate-resistant acid phosphatase (TRAP) staining

Differentiated osteoclasts were identified via TRAP staining using a commercially available kit (Sigma–Aldrich 387A, St. Louis, MO) following the manufacturer's protocol. TRAP-positive osteoclasts were counted within 400 ​μm of the cement line in the osteochondral junction and divided by the length of the subchondral bone to give osteoclast density, expressed as TRAP-positive cells per millimeter. One dark-purplish or reddish cell with ≥3 nuclei was recorded as one osteoclast [10].

### 2.7 Image analysis

Histological scoring and quantification were performed using an all-in-one fluorescence microscope BZ-X800 with analysis using BZ-X800 Analyzer (Keyence), by a single observer (KA) blinded to the details of the sections.

### 2.8 Statistical analysis

All statistical analyses were performed using JMP 10 (SAS Ins. Cary, NC, USA) and IBM SPSS 26.0 (IBM Corp. Armonk, NY, USA). Osteoarthritis bone scores, OARSI grade and Mankin scores were analyzed using Friedman tests with a post-hoc Bonferroni test. Comparison of NGF expression in the subchondral bone and osteochondral channel, channel density, and osteoclast density was done using one-way repeated measures ANOVA with a post-hoc Tukey test among the three osteochondral tissue types. Comparison of NGF expression and osteoclast density between MTP bone with and without cysts was done using unpaired *t*-test. Spearman rank correlation coefficient assessed associations between NGF expression and osteoclast density. Differences with *p* ​< ​0.05 were considered statistically significant. In a previous study [10] comparing samples from symptomatic and asymptomatic knee OA, 0.23 osteoclasts mm^−1^ was considered a clinically significant difference. Therefore, a difference of 0.23 osteoclasts mm^−1^ was used as the threshold here. To demonstrate a difference of 0.23 osteoclasts mm^−1^ with 90% confidence and *α* ​= ​0.05, it was determined that 20 patients were needed.

## 3 Results

**Fig. 2:**
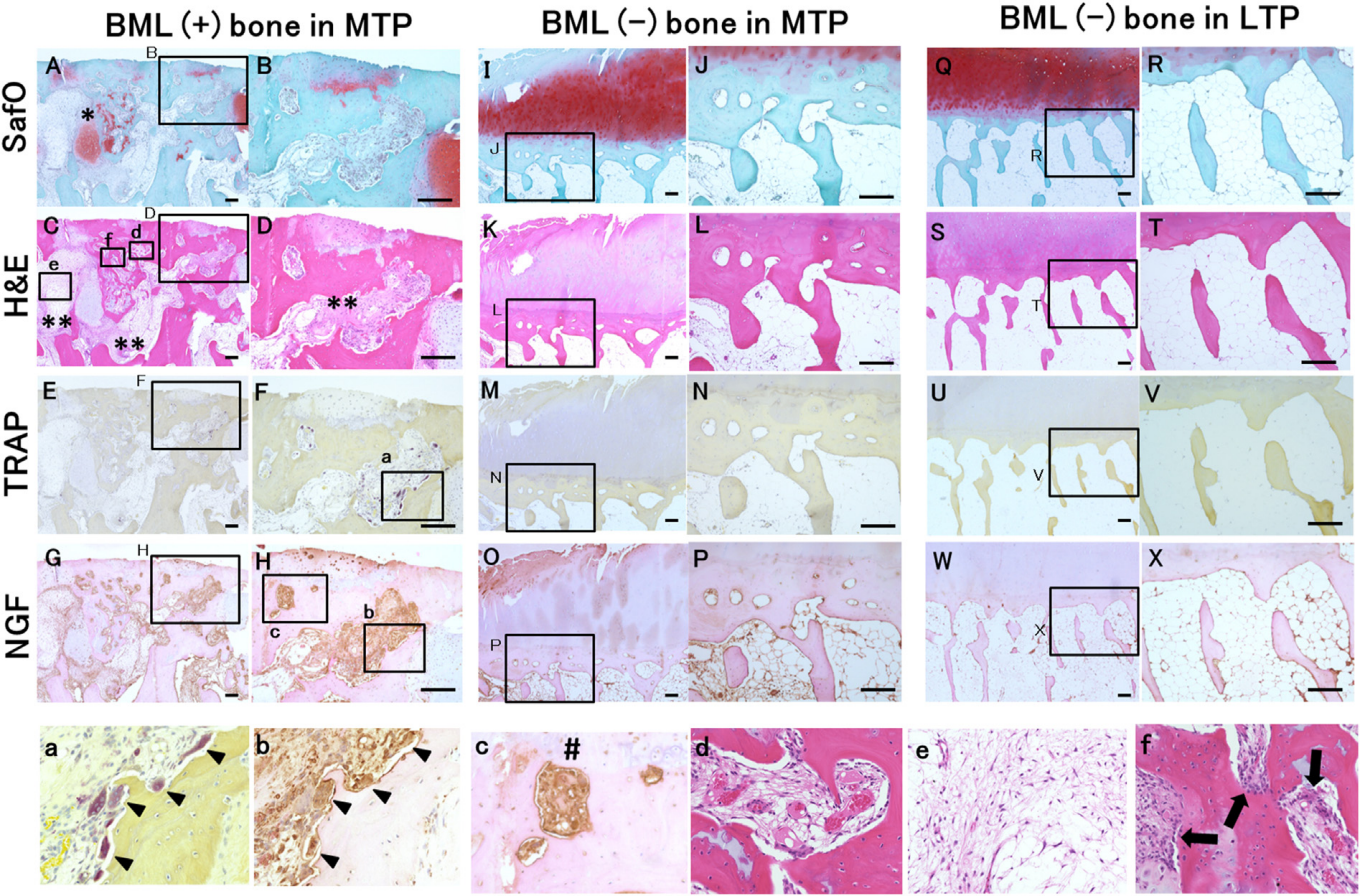
**Histopathologic features in BML ​^+^ ​MTP bone, BML^−^ MTP bone and BML^−^ LTP bone** Adjacent sections were stained with Safranin O and fast green, H&E, TRAP, and were processed for NGF. Sections from BML^+^ MTP bone show severe damage on cartilage surface with loss of proteoglycans, thickened trabeculae, cartilage islands (appears pink on Safranin O stain (A; asterisk), cyst-like lesions (C, D; double asterisk), vascular proliferation (d) bone marrow fibroses (C and e), cellular infiltration (C and f) and increased presence of osteoclasts (F, a and b). NGF immunoreactivity was detected in multinucleate TRAP positive osteoclasts adherent to bone (a and b), fibroblast-like cell in fibrotic connective tissue (H), mononuclear cells (b) and cartilage islands in bone marrow space (G). BML^+^ MTP bone exhibits an NGF-positive osteochondral channel (c; sharp) while BML^−^ MTP bones exhibits an NGF-negative osteochondral channel (N). Cyst-like lesions frequently observed in BML ​+ ​MTP bone (C, D; double asterisk), and contained fibrous connective tissue (e), cartilage islands (A; asterisk) and TRAP positive osteoclasts which increased mainly on the surface of cysts (a). SafO: Safranin O and fast green, H&E: hematoxylin and eosin, SafO: Safranin O and fast green, TRAP: tartrate-resistant acid phosphatase, NGF: nerve growth factor, MTP: medial tibia plateau, LTP: lateral tibial plateau. Bars ​= ​200 ​μm

The demographic details of the study participants are presented in Table 1, and the histological characteristics in Fig. 2 and Table 2. The OA bone score, OARSI OA cartilage histopathology assessment system and Mankin score of BML^+^ MTP bone were significantly higher than those of BML^−^ MTP and BML^−^ LTP bones (*p* ​< ​0.01 versus BML− MTP and BML− LTP bones); further, these scores were significantly higher in BML^−^ MTP bone than in BML^−^ LTP bone (*p* ​< ​0.01 versus BML− LTP bone) (Table 2).

**Table 1:** Subject demographics.

Age (years)	77 (75, 80)
BMI (kg/m^2^)	26 (25, 28)
Female, number of patients	17
WOMAC score	
Pain score, 0-20	8 (6,11)
Stiffness score, 0-8	3 (3, 4)
Function score, 0-68	25 (23, 32)
Pain VAS during walking (mm), 0-100	50 (45, 73)
Pain VAS at rest (mm), 0-100	10 (0, 30)

Data displayed as mean (95% confidence interval (CI)) for age and BMI, and median (Interquartile range (IQR)) for WOMAC score and Pain VAS. BMI: body mass index, WOMAC: Western Ontario and McMaster Universities Arthritis Index.

**Table 2:** Histologic characteristics.

	BML^+^ MTP bone	BML^−^ MTP bone	BML^−^ LTP bone
Osteoarthritis bone score, 0–7	6 (5, 7)^a^	3 (2, 4)^c^	0 (0, 0)
Cysts, 0–1	1 (0, 1)^a^	0 (0, 0)	0 (0, 0)
Fibrotic connective tissue, 0–1	1 (1, 1)^c^	1 (1, 1)^c^	0 (0, 0)
Blood vessels, 0–1	1 (1, 1)^a^	0 (0, 0)	0 (0, 0)
Cartilage islands, 0–1	1 (0, 1)^a^	0 (0, 0)	0 (0, 0)
Trabeculae thickened, 0–1	1 (1, 1)^a^	1 (0, 1)^a^	0 (0, 0)
Tidemark integrity, 0–1	1 (1, 1)^c^	1 (1, 1)^c^	0 (0, 0)
Cellular infiltrates, 0–1	1 (1, 1)^c^	0.5 (0, 1)^c^	0 (0, 0)
OA cartilage pathology (OARSI grade)	6 (6, 6)^a^	4 (4, 5)^c^	2 (2, 2)
Total Mankin score, 0–14	14 (14, 14)^a^	7 (6, 9)^c^	4 (3, 5)
Cartilage surface integrity, 0–6	6 (6, 6)^a^	3 (3, 3)^c^	1 (1, 1)
Chondrocyte appearance, 0–3	3 (3, 3)^a^	1 (1, 2)	1 (1, 1)
Tidemark integrity, 0–1	1 (1, 1)^a^	0 (0, 1)	0 (0, 0)
Proteoglycan loss, 0–4	4 (4, 4)^a^	2 (2, 3)^c^	1 (1, 2)
Osteochondral channel density, mm	2 (2, 3)^b^	2 (2, 2)	1 (1, 2)

Data displayed as median (IQR) for osteoarthritis bone score, OARSI grade and Mankin score, and mean (95% CI) for osteochondral channel density and % of NGF positive channel. MTP: medial tibia plateau, LTP: lateral tibial plateau, NGF: nerve growth factor.

a P ​< ​0.01 versus BML^−^ MTP bone and BML^−^ LTP bone.

b P ​< ​0.05 versus BML^−^ LTP bone.

c P ​< ​0.01 versus BML^−^ LTP bone.

**Fig. 3:**
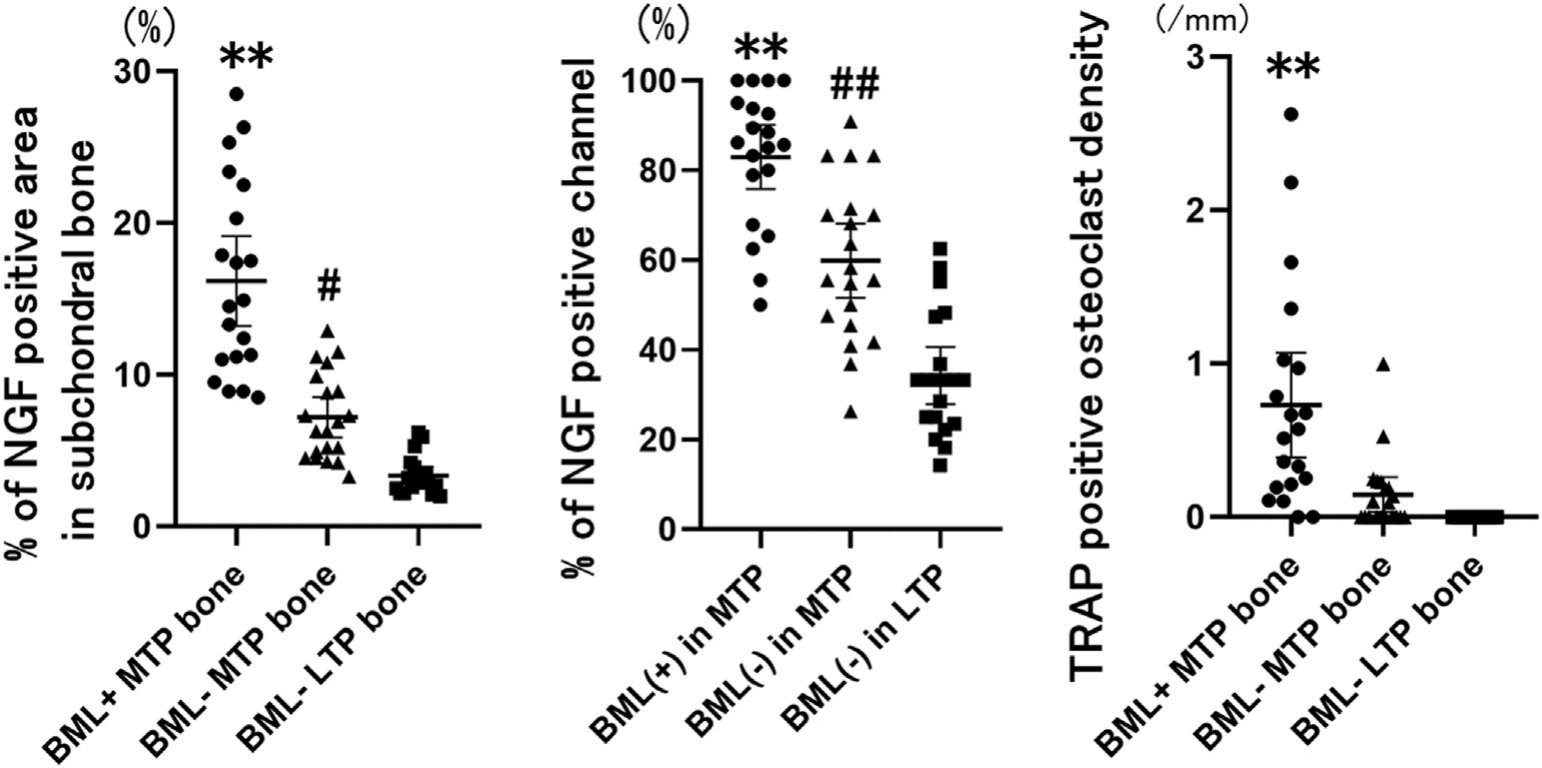
**NGF expression in the subchondral bone marrow space and the osteochondral channel, and TRAP–positive osteoclasts in BML+MTP bone, BML**^**−**^**MTP bone and BML**^**−**^**LTP bone.** Each symbol represents an individual sample; bars show the mean and 95% CI. TRAP: tartrate-resistant acid phosphatase, NGF: nerve growth factor, MTP: medial tibia plateau, LTP: lateral tibial plateau. ∗∗P ​< ​0.01 versus BML^−^ MTP bone and BML^−^ LTP bone. #P ​< ​0.05 versus BML^−^ LTP bone. ##P ​< ​0.01 versus BML− LTP bone.

In the MTP samples, NGF immunoreactivity was detected in multinucleate osteoclast-like cells adherent to the bone, in fibroblast-like cells in fibrotic connective tissue, and mononuclear cells in the bone marrow space in the subchondral bone (Fig. 2). The NGF-immunoreactive cells were also found in osteochondral channels (Fig. 2). The NGF-positive area in the subchondral bone marrow space and NGF expression within the osteochondral channel were significantly higher in BML^+^ MTP bone than in BML^−^ MTP and BML^−^ LTP bones (*p* ​< ​0.01), and the NGF-positive area and channel were significantly higher in BML^−^ MTP bone than in BML^−^ LTP bone (NGF-positive area: *p* ​< ​0.05, NGF-positive channel: *p* ​< ​0.01). For subchondral MTP bone, the mean differences in NGF-expressing area and percentage of NGF-positive channels between bone with and without BMLs were 9.0% (95% confidence interval [CI]: 5.9–12.1%) and 23.1% (95% CI: 11.3–35.0%), respectively (Table 2, Fig. 3).

TRAP-positive multinucleated osteoclasts were observed on the surface of the subchondral trabecular bone (Fig. 2). Subchondral bone osteoclast density was significantly higher in BML^+^ MTP bone than in BML^−^ MTP and in BML^−^ LTP bones (*p* ​< ​0.01). For subchondral MTP bone, the mean difference in osteoclast density between the groups with and without BML was 0.6 osteoclasts per mm (95% CI: 0.3–0.9 osteoclasts per mm). No significant difference in osteoclast density was observed between BML^−^ MTP and BML^−^ LTP bones in the LTP (Fig. 3).

**Fig. 4:**
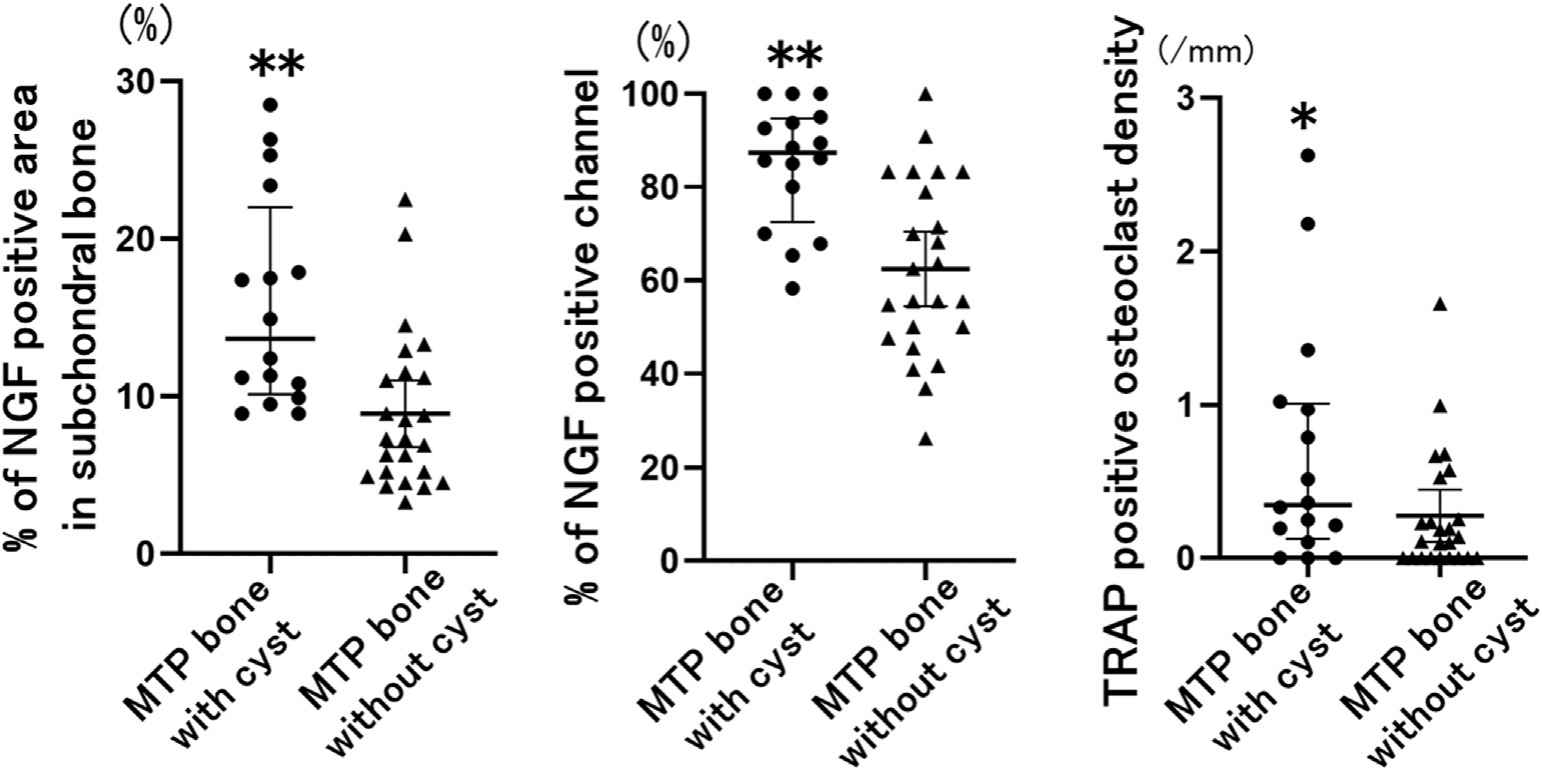
**NGF expression in the subchondral bone marrow space and the osteochondral channel, and TRAP–positive osteoclasts in MTP bone with and without cysts.** Each symbol represents an individual sample; bars show the mean and 95% CI. TRAP: tartrate-resistant acid phosphatase, NGF: nerve growth factor, MTP: medial tibia plateau, LTP: lateral tibial plateau. ∗P ​< ​0.05 versus MTP bone without cyst. ∗∗P ​< ​0.01 versus MTP bone without cyst.

Based on the analyses of the relationship of NGF expression and osteoclast density in subchondral MTP bone, there were significant correlations between NGF-positive area and channel, and osteoclast density (area: Spearman's r ​= ​0.50, 95% CI: 0.24–0.71; channel: Spearman's r ​= ​0.52, 95% CI: 0.23–0.70). Cyst-like lesions are frequently observed in BML ​+ ​MTP bone, and TRAP-positive osteoclasts which increase mainly on the surface of the cysts (Fig. 2). Based on analyses of associations between NGF expression and osteoclast density with subchondral bone cysts, MTP bone with cysts had a significantly higher percentage of NGF-positive area and channels, as well as greater osteoclast density, compared to MTP bone without cysts (NGF-positive area and channel: *p* ​< ​0.01, osteoclast density: *p* ​< ​0.05). The differences in the percentage of NG-positive area and channel were 7.0% (95% CI: 3.2–10.7%) and 22.4% (95% CI: 11.3–33.4%), respectively, and the difference of osteoclast density was 0.4/mm (95% CI: 0.02–0.8/mm) (Fig. 4).

## 4 Discussion

In this study, we demonstrated increased NGF expression and osteoclast density in subchordal bone with BMLs compared with those in subchondral bone without BMLs in the same individual. NGF^-^positive cells and osteoclasts in subchondral bone with BMLs can induce sensitization of bone afferent neurons, and changes in subchondral bone quality may increase mechanosensitivity. BML-like histopathological changes are associated with subchondral innervation [13]. For subchondral bone with BMLs, we observed elevated NGF expression in bone marrow space and osteochondral channel, and levels of osteoclasts, which can promote nerve growth; these findings advance the earlier findings on innervation in subchondral bone in knee OA. BMLs may contribute directly to OA pain, and our results contribute to the understanding of the cellular and molecular factors that mediate the association between subchondral BMLs and knee OA pain.

Our findings showed elevated NGF expression in the subchondral bone marrow space and at the osteochondral junction in patients with BML. Furthermore, the elevated NGF expression was also associated with subchondral bone cysts. Our current results extend findings from a previous study [12], wherein subchondral BMLs with cysts were shown to exhibit increased osteoclastogenesis and nerve distribution. Increased NGF immunoreactivity was detected in multinucleate osteoclast-like cells adherent to the bone, fibroblast-like cells in the fibrotic connective tissue, mononuclear cells, and cartilage islands in the subchondral bone. NGF may directly activate sensory neurons that express TrkA and modulate the expression of TrkA or the p75 receptor [16]. Anti-NGF antibodies can reduce OA pain, indicating the importance of NGF in pain generation [17,18]. Increased numbers of NGF-immunoreactive cells in the osteochondral channels and bone marrow space could contribute to OA pain by increasing colocalized sensory nerve activity. Microarray studies in OA BML tissues have revealed the upregulation of genes implicated in neurogenesis [9]. Anti-NGF monoclonal antibodies provide significant improvements in pain and function; however, the use of tanezumab leads to more frequent adverse events, namely abnormal peripheral sensation and rapidly progressive OA, than the use of nonsteroidal anti-inflammatory drugs [19,20].

Our results showed an increase in the number of multinucleate osteoclasts, which can resorb adjacent bone tissue in the subchondral bone with BML and MTP with cysts, consistent with a previous study [12]. However, in the abovementioned study, multinucleated activated osteoclasts were not observed, and the results were influenced by systemic differences in bone metabolism, as the study compared individuals with and without BMLs. Our study showed that osteoclasts are a source of NGF, which could sensitize primary afferents in the subchondral bone, and the osteoclast density was significantly correlated with NGF expression. Osteoclasts also secrete netrin-1 and protons to activate sensory nerves [21]. As stated above, osteoclasts may play a key role in BML-related bone pain. In clinical studies, high serum concentrations of TRAP-5b, an indicator of osteoclast number, are associated with subchondral osteoclast density, OA pain, and a poor pain prognosis [22]. Studies of the effects of osteoclast inhibitors, such as bisphosphonates, denosumab, and strontium ranelate [23], have revealed joint pain reduction in people with knee OA. Zoledronic acid, a bisphosphonate, reduced knee pain and BML size in patients with OA [24], although findings from a meta-analysis of randomized controlled trials did not support the analgesic effects of bisphosphonates in knee OA [25]. Our data suggest that there are multiple mechanisms underlying OA knee pain with multiple sources and that targeting osteoclasts has clinically important benefits only in cases of subchondral BML, in which osteoclast activity is the predominant driver of pain. Further studies are needed to develop treatments targeting BML-related pain in patients with knee OA.

Subchondral cyst-like lesions are sometimes observed in knee OA and commonly develop within regions of BML and adjacent to cartilage abnormalities [26]. Previous histological studies showed subchondral cysts containing fibrous connective tissue, osteoblasts, adipocytes [27], and TRAP-positive cells [12], which is consistent with our results. Another study demonstrated that subchondral BMLs with cysts had increased osteoclastogenesis compared with subchondral BMLs without cysts. However, in this study, there were no significant differences in osteoclast density between subchondral BMLs with and without cysts, possibly due to the small sample size (data not shown). Therefore, we compared osteoclast density and NGF expression between MTP bone with and without cysts. We found that MTP bones with cysts had a significantly higher NGF expression (reported for the first time in this study) and osteoclast density, compared to MTP bones without cysts. In a large-scale observational study, MRI-assessed subchondral cysts were associated with the development of knee pain in knee OA [28]. Our results may explain one mechanism by which subchondral cysts are related to knee pain in knee OA.

Recently, interventions directly targeting subchondral bone BMLs have been attempted. Subchondroplasty, which utilizes an injectable synthetic calcium phosphate bone void filler to treat BMLs, significantly improved pain relief and knee function [29]. Extracorporeal shockwave therapy achieved good results in treating the treatment of BMLs [30,31]. Injecting mesenchymal stem cells into the subchondral bone can potentially treat BMLs [32], while the evidence for these interventions remains limited. However, our histological results, which reveal increased levels of pain-related factors in subchondral BMLs, provide support for interventions and treatments directly targeting BMLs.

A previous study has suggested that subchondral BMLs might mediate mechanically induced pain, such as during weight-bearing activity [7]. Biomechanical factors may contribute reciprocally to the pathogenesis of subchondral BMLs. Increased mechanical load due to malalignment of the knee joint is a risk factor for the occurrence or enlargement of femorotibial joint subchondral BMLs [33,34]. The meniscus and cartilage can act as shock absorbers that protect the subchondral bone from overload. The increase in subchondral BML size with worsening meniscal pathology [35,36] or cartilage loss [35,37] may be mediated by changes in biomechanical forces through the subchondral bone. Therefore, biomechanical unloading is an attractive treatment option for the treatment of subchondral BMLs. High tibial osteotomy, which alters the load distribution between the medial and lateral compartments of the knee, reduces BML size in the femorotibial compartments [38]. In patients with painful patellofemoral OA, patellofemoral bracing reduces the number of BMLs in the patellofemoral joint compartments [39].

This study has several limitations. While tissue samples with BMLs were obtained from the weight-bearing part of the MTP, the findings might differ for other joint regions such as the femoral condyles. All subjects included in this study had BMLs in the medial tibial plateaus and subjects without BMLs in the medial tibial plateaus were not evaluated. However, patients with knee OA who exhibit BMLs are known to experience greater pain compared to those without BMLs, therefore we focused on pain-related histological changes of BMLs. Osteoclast activity itself was not examined in this study; however, cells with ≥3 nuclei were counted as a single osteoclast to estimate the number of active osteoclasts, as resorption activity has been shown under some circumstances to correlate with the number of nuclei present [40]. Despite these limitations, these limitations, our study clarifies how subchondral BMLs may cause joint pain.

## 5 Conclusions

Increased NGF expression and osteoclast density appear to have important associations with bone pain in knee OA with BMLs. This study will contribute to understanding the pathophysiology of subchondral BMLs in OA and identifying new therapeutic targets for the management of BML-related OA pain.

## Funding

This study was supported by Grants-in-Aid for Scientific Research (KAKENHI; 20K18001).

## Ethics approval

This study was approved by the Kochi University Research Ethics Committee (IRB:31–74).

## Consent to participate

Informed consent was obtained from all the patients.

## Consent for publication

All authors agreed to the publication of this manuscript.

## Author contributions

All authors approved the final version of the manuscript. K.A. has full access to all of the study data and takes responsibility for the integrity of the data and the accuracy of the data analysis. K.A. designed the study, performed histological processing, analyzed and interpreted the results, and wrote the manuscript. N.S., H.W., S.D., and M.I. interpreted the results. M.I. supervised the study.

## Declaration of competing interest

All authors declare no competing interests.

## References

[1] Stahl R., Jain S.K., Lutz J., Wyman B.T., Le Graverand-Gastineau M.P., Vignon E. (2011). Osteoarthritis of the knee at 3.0 T: comparison of a quantitative and a semi-quantitative score for the assessment of the extent of cartilage lesion and bone marrow edema pattern in a 24-month longitudinal study. Skeletal Radiol..

[2] Lo G.H., McAlindon T.E., Niu J., Zhang Y., Beals C., Dabrowski C. (2009). Bone marrow lesions and joint effusion are strongly and independently associated with weight-bearing pain in knee osteoarthritis: data from the osteoarthritis initiative. Osteoarthritis Cartilage.

[3] Driban J.B., Price L., Lo G.H., Pang J., Hunter D.J., Miller E. (2013). Evaluation of bone marrow lesion volume as a knee osteoarthritis biomarker--longitudinal relationships with pain and structural changes: data from the Osteoarthritis Initiative. Arthritis Res. Ther..

[4] Hunter D.J., Guermazi A., Lo G.H., Grainger A.J., Conaghan P.G., Boudreau R.M. (2011). Evolution of semi-quantitative whole joint assessment of knee OA: MOAKS (MRI Osteoarthritis Knee Score). Osteoarthritis Cartilage.

[5] Walsh D.A., Sofat N., Guermazi A., Hunter D.J. (2023). Osteoarthritis bone marrow lesions. Osteoarthritis Cartilage.

[6] Roemer F.W., Collins J.E., Neogi T., Crema M.D., Guermazi A. (2020). Association of knee OA structural phenotypes to risk for progression: a secondary analysis from the Foundation for National Institutes of Health Osteoarthritis Biomarkers study (FNIH). Osteoarthritis Cartilage.

[7] Aso K., Shahtaheri S.M., McWilliams D.F., Walsh D.A. (2021). Association of subchondral bone marrow lesion localization with weight-bearing pain in people with knee osteoarthritis: data from the Osteoarthritis Initiative. Arthritis Res. Ther..

[8] Hunter D.J., Gerstenfeld L., Bishop G., Davis A.D., Mason Z.D., Einhorn T.A. (2009). Bone marrow lesions from osteoarthritis knees are characterized by sclerotic bone that is less well mineralized. Arthritis Res. Ther..

[9] Kuttapitiya A., Assi L., Laing K., Hing C., Mitchell P., Whitley G. (2017). Microarray analysis of bone marrow lesions in osteoarthritis demonstrates upregulation of genes implicated in osteochondral turnover, neurogenesis and inflammation. Ann. Rheum. Dis..

[10] Aso K., Shahtaheri S.M., Hill R., Wilson D., McWilliams D.F., Walsh D.A. (2019). Associations of symptomatic knee osteoarthritis with histopathologic features in subchondral bone. Arthritis Rheumatol..

[11] Morgan M., Nazemian V., Harrington K., Ivanusic J.J. (2022). Mini review: the role of sensory innervation to subchondral bone in osteoarthritis pain. Front. Endocrinol..

[12] Zhou F., Han X., Wang L., Zhang W., Cui J., He Z. (2022). Associations of osteoclastogenesis and nerve growth in subchondral bone marrow lesions with clinical symptoms in knee osteoarthritis. J Orthop Translat.

[13] Koushesh S., Shahtaheri S.M., McWilliams D.F., Walsh D.A., Sheppard M.N., Westaby J. (2022). The osteoarthritis bone score (OABS): a new histological scoring system for the characterization of bone marrow lesions in osteoarthritis. Osteoarthritis and cartilage/OARS. Osteoarthritis Research Society.

[14] Pritzker K.P., Gay S., Jimenez S.A., Ostergaard K., Pelletier J.P., Revell P.A. (2006). Osteoarthritis cartilage histopathology: grading and staging. Osteoarthritis Cartilage.

[15] Shu S.Y., Ju G., Fan L.Z. (1988). The glucose oxidase-DAB-nickel method in peroxidase histochemistry of the nervous system. Neurosci. Lett..

[16] Pezet S., McMahon S.B. (2006). Neurotrophins: mediators and modulators of pain. Annu. Rev. Neurosci..

[17] Sanga P., Katz N., Polverejan E., Wang S., Kelly K.M., Haeussler J. (2013). Efficacy, safety, and tolerability of fulranumab, an anti-nerve growth factor antibody, in the treatment of patients with moderate to severe osteoarthritis pain. Pain.

[18] Lane N.E., Schnitzer T.J., Birbara C.A., Mokhtarani M., Shelton D.L., Smith M.D. (2010). Tanezumab for the treatment of pain from osteoarthritis of the knee. N. Engl. J. Med..

[19] Neogi T., Hunter D.J., Churchill M., Shirinsky I., White A., Guermazi A. (2022). Observed efficacy and clinically important improvements in participants with osteoarthritis treated with subcutaneous tanezumab: results from a 56-week randomized NSAID-controlled study. Arthritis Res. Ther..

[20] Hochberg M.C., Carrino J.A., Schnitzer T.J., Guermazi A., Walsh D.A., White A. (2021). Long-term safety and efficacy of subcutaneous tanezumab versus nonsteroidal antiinflammatory drugs for hip or knee osteoarthritis: a randomized trial. Arthritis Rheumatol..

[21] Zhu S., Zhu J., Zhen G., Hu Y., An S., Li Y. (2019). Subchondral bone osteoclasts induce sensory innervation and osteoarthritis pain. J. Clin. Invest..

[22] Nwosu L.N., Allen M., Wyatt L., Huebner J.L., Chapman V., Walsh D.A. (2017). Pain prediction by serum biomarkers of bone turnover in people with knee osteoarthritis: an observational study of TRAcP5b and cathepsin K in OA. Osteoarthritis and cartilage/OARS. Osteoarthritis Research Society.

[23] Reginster J.Y., Badurski J., Bellamy N., Bensen W., Chapurlat R., Chevalier X. (2013). Efficacy and safety of strontium ranelate in the treatment of knee osteoarthritis: results of a double-blind, randomized placebo-controlled trial. Ann. Rheum. Dis..

[24] Laslett L.L., Dore D.A., Quinn S.J., Boon P., Ryan E., Winzenberg T.M. (2012). Zoledronic acid reduces knee pain and bone marrow lesions over 1 year: a randomized controlled trial. Ann. Rheum. Dis..

[25] Vaysbrot E.E., Osani M.C., Musetti M.C., McAlindon T.E., Bannuru R.R. (2018). Are bisphosphonates efficacious in knee osteoarthritis? A meta-analysis of randomized controlled trials. Osteoarthritis and cartilage/OARS. Osteoarthritis Research Society.

[26] Carrino J.A., Blum J., Parellada J.A., Schweitzer M.E., Morrison W.B. (2006). MRI of bone marrow edema-like signal in the pathogenesis of subchondral cysts. Osteoarthritis Cartilage.

[27] Pouders C., De Maeseneer M., Van Roy P., Gielen J., Goossens A., Shahabpour M. (2008). Prevalence and MRI-anatomic correlation of bone cysts in osteoarthritic knees. AJR Am. J. Roentgenol..

[28] Perry T.A., O'Neill T.W., Tolstykh I., Lynch J., Felson D.T., Arden N.K. (2022). Magnetic resonance imaging-assessed subchondral cysts and incident knee pain and knee osteoarthritis: data from the multicenter osteoarthritis study. Arthritis Rheumatol..

[29] Pasqualotto S., Sgroi A.V., Causero A., Di Benedetto P., Zorzi C. (2019). Subchondroplasty in the treatment of bone marrow lesions of the knee: preliminary experience on first 15 patients. Joints.

[30] Gao F., Sun W., Li Z., Guo W., Wang W., Cheng L. (2015). Extracorporeal shock wave therapy in the treatment of primary bone marrow edema syndrome of the knee: a prospective randomized controlled study. BMC Muscoskel. Disord..

[31] Vitali M., Naim Rodriguez N., Pedretti A., Drossinos A., Pironti P., Di Carlo G. (2018). Bone marrow edema syndrome of the medial femoral condyle treated with extracorporeal shock wave therapy: a clinical and MRI retrospective comparative study. Arch. Phys. Med. Rehabil..

[32] Hernigou P., Delambre J., Quiennec S., Poignard A. (2021). Human bone marrow mesenchymal stem cell injection in subchondral lesions of knee osteoarthritis: a prospective randomized study versus contralateral arthroplasty at a mean fifteen year follow-up. Int. Orthop..

[33] Beckwée D., Vaes P., Shahabpour M., Muyldermans R., Rommers N., Bautmans I. (2015). The influence of joint loading on bone marrow lesions in the knee: a systematic review with meta-analysis. Am. J. Sports Med..

[34] Lim Y.Z., Wang Y., Wluka A.E., Davies-Tuck M.L., Hanna F., Urquhart D.M. (2014). Association of obesity and systemic factors with bone marrow lesions at the knee: a systematic review. Semin. Arthritis Rheum..

[35] Antony B., Venn A., Cicuttini F., March L., Blizzard L., Dwyer T. (2016). Correlates of knee bone marrow lesions in younger adults. Arthritis Res. Ther..

[36] Englund M., Guermazi A., Roemer F.W., Yang M., Zhang Y., Nevitt M.C. (2010). Meniscal pathology on MRI increases the risk for both incident and enlarging subchondral bone marrow lesions of the knee: the MOST Study. Ann. Rheum. Dis..

[37] Hunter D.J., Zhang Y., Niu J., Goggins J., Amin S., LaValley M.P. (2006). Increase in bone marrow lesions associated with cartilage loss: a longitudinal magnetic resonance imaging study of knee osteoarthritis. Arthritis Rheum..

[38] Choi H.G., Kim J.S., Yoo H.J., Jung Y.S., Lee Y.S. (2021). The fate of bone marrow lesions after open wedge high tibial osteotomy: a comparison between knees with primary osteoarthritis and subchondral insufficiency fractures. Am. J. Sports Med..

[39] Callaghan M.J., Parkes M.J., Hutchinson C.E., Gait A.D., Forsythe L.M., Marjanovic E.J. (2015). A randomized trial of a brace for patellofemoral osteoarthritis targeting knee pain and bone marrow lesions. Ann. Rheum. Dis..

[40] Boissy P., Saltel F., Bouniol C., Jurdic P., Machuca-Gayet I. (2002). Transcriptional activity of nuclei in multinucleated osteoclasts and its modulation by calcitonin. Endocrinology.

