# Supplementary material for: Increased nerve growth factor expression and osteoclast density are associated with subchondral bone marrow lesions in osteoarthritic knees
Source: Osteoarthr Cartil Open. 2024 Jul 23;6(3):100504. doi: 10.1016/j.ocarto.2024.100504 (PMC11340585; doi:10.1016/j.ocarto.2024.100504)
Supplement: Multimedia component 1 [file mmc1.docx]

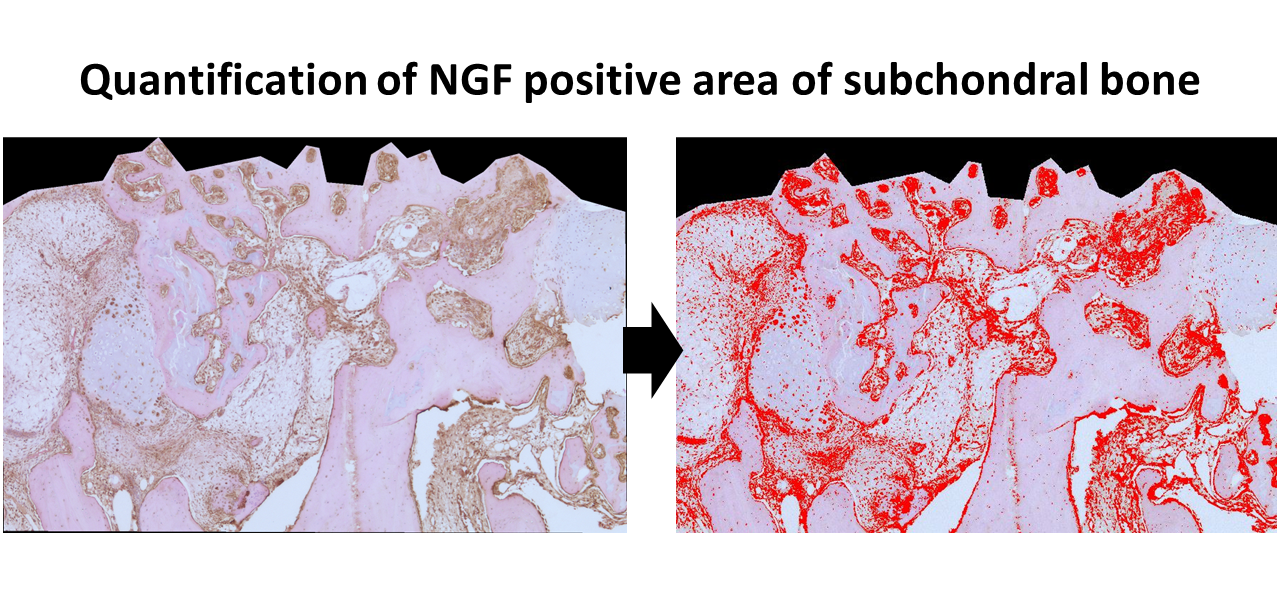


**Supplementary figure 1. Quantification of NGF expression**

NGF positive staining was differentiated from background by thresholding the image according to hue in order to create a mask. The area of positive staining (red area) was automatically measured. The fractional area was determined as the percentage of positive area for NGF immunoreactivity (red area) within the area of ROIs (area of subchondral bone marrow space).


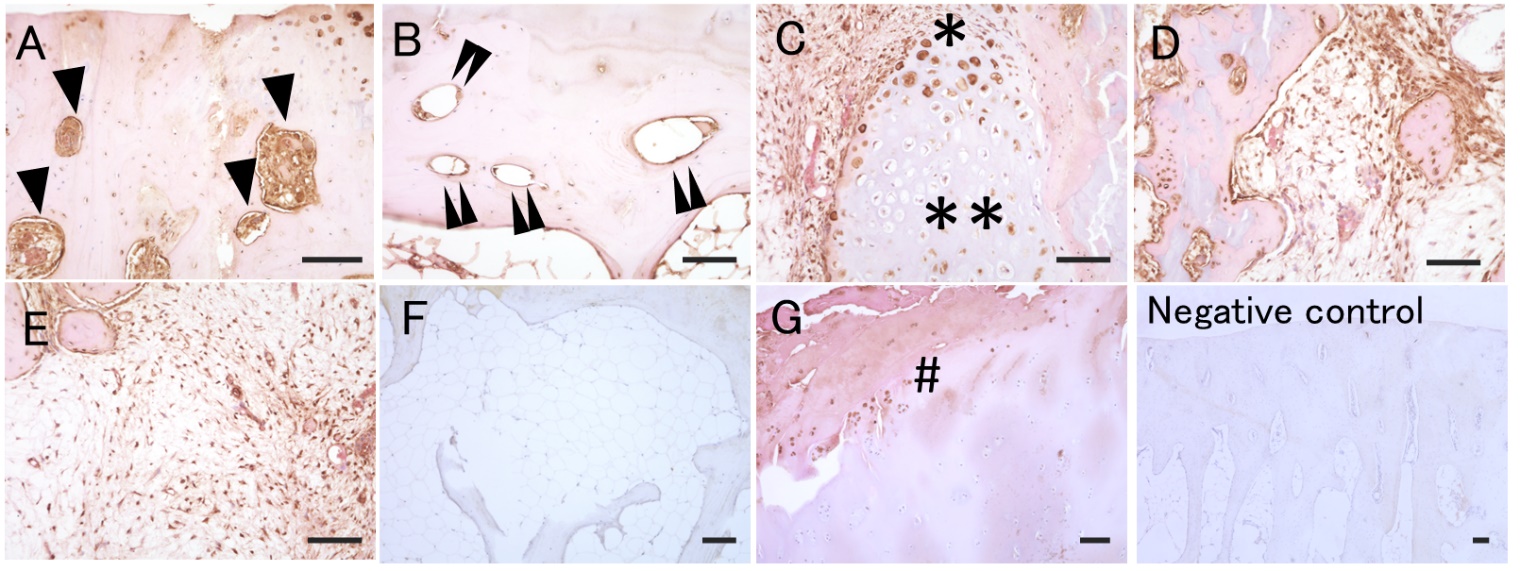


**Supplementary figure 2. NGF expression in osteochondral tissue**

NGF immunoreactivity was detected in some osteochondral channels (A; arrowhead), in some chondrocytes in cartilage islands (C; asterisk), in mononuclear cells in the bone marrow space (D), in fibroblast-like cells in fibrotic connective tissue (E), and in degenerated chondrocytes in cartilage (G; sharp). On the other hand, some osteochondral channels (B; double arrowhead) which located mainly BML- MTP and LTP bone, in some chondrocytes in cartilage islands (C; double asterisk) and adipose tissue were NGF negative (F).

NGF: nerve growth factor, MTP: medial tibia plateau, LTP: lateral tibial plateau. Bars = 200μm
